# Reply to: Machine-learning prediction of hosts of novel coronaviruses requires caution as it may affect wildlife conservation

**DOI:** 10.1038/s41467-022-32747-6

**Published:** 2022-09-12

**Authors:** Marcus S. C. Blagrove, Matthew Baylis, Maya Wardeh

**Affiliations:** 1 Department of Evolution, Ecology, and Behaviour, Institute of Infection, Veterinary & Ecological Sciences, University of Liverpool, Biosciences Building, Crown Street, Liverpool, L69 7ZB UK; 2 Department of Livestock and One Health, Institute of Infection, Veterinary & Ecological Sciences, University of Liverpool, Liverpool Science Park IC2 Building, 146 Brownlow Hill, Liverpool, L3 5RF UK; 3 Health Protection Research Unit in Emerging and Zoonotic Infections, University of Liverpool, Liverpool, L69 7BE UK; 4 Department of Mathematical Sciences, University of Liverpool, Peach Street, Liverpool, L69 7ZL UK

**Keywords:** Viral evolution, Viral reservoirs, Machine learning

**replying to** Rasmussen et al. *Nature Communications* 10.1038/s41467-022-32746-7 (2022)

We appreciate the comments made by Rasmussen et al.^1^ regarding our recent study, Wardeh et al.^2^, which uses machine learning to predict hosts that may become co-infected with multiple different coronaviruses, and hence may be the site of recombination and generation of novel future strains. We take this opportunity to, first, summarise the methodology in our study, and, second, to respond to their comments. We also discuss general limitations of the available data, their utilisation, and future avenues of research to mitigate some of these limitations. Rasmussen et al.^1^. do focus somewhat on a tabloid article published in the ‘Daily Star’; we take this opportunity to state that none of the authors of Wardeh et al.^2^, were consulted or had any involvement with the quoted article. We would also like to reiterate our original article^2^ in stating that our predictions are meant to inform surveillance and policy makers, and we in no way suggested that public individuals should take any action.

To summarise the methods in our study:^2^ In order to identify mammals which are potentially susceptible to two or more coronaviruses, we developed a computational framework to predict associations between known coronaviruses and potential mammalian hosts. To achieve this task, we separated the prediction problem into three perspectives: that of the mammal, the coronavirus, and the network linking known coronaviruses with their hosts. In each perspective, we implemented several similarity-based learners (Supplementary Table 1 provides a comprehensive overview). Each learner computed a score, termed confidence (value between 0 and 1), for each possible coronavirus-mammal association (over 300,000 potential associations). For instance, in the mammalian perspective, the score is closer to 1 where the focal mammal is more similar to known hosts of the focal virus, and also more dissimilar to mammals not known to be hosts of the focal virus. Conversely, the score is closer to 0 if the mammal is more similar to mammals not known to be hosts than it is to known hosts of the focal virus. Importantly, we calculated similarities for each learner based on a distinct factor, or group of factors (Supplementary Table 1). Where multiple factors were considered, we utilised similarity network fusion (SNF)^3^ technique to integrate the similarities computed for each factor into one so not to inflate the number of learners.

As our confidence scores (Supplementary Table 1) were computed based on a specific factor (e.g., habitat utilisation) or group of factors (e.g., predicted secondary structure) in a single perspective, we “blended” these scores by utilising a GBM-based ensemble. The ensemble integrated all generated confidence scores (12 in total for every potential association), in a non-linear way, to generate final predictions. Our ensemble comprised 100 replicate models, each trained with a subset of the computed confidence scores, and subsequently generated predictions over the whole set of potential associations. These predictions were produced as probabilities (value between 0 and 1), final predictions were computed by taking the mean of the 100 models, and three cut-off points were used in generating the results: 0.5, 0.75, and 0.9821 – whereby a coronavirus-mammal association is predicted to be feasible if mean probability exceeded (or equalled) the given cut-off (further details about these cut-offs and reasons for selection can be found in Wardeh et al.^2^).

The utilisation of multiple cut-offs allowed us to present a range of predictions, on one hand, the lowest cut-off minimises the number of false negative, whereas the highest cut-off minimises number of potentially false positive. Due to the large number of potential associations (> 300,000), it is infeasible to perform *post-hoc* validation of each of them. Confusion matrices allow us to illustrate the trade-offs between false negatives and potentially false positives at varying cut-offs; Supplementary Figs. 10–12 in ref. 2 visualise confusion matrices resulting from 20 tests (15% of known associations omitted in each). We deliberately provided all of our results throughout the study to all three of these cut-offs, and indeed, SARS-CoV-2 association with the European hedgehog is not predicted at our more conservative cut-off. We believe that all results should be presented, and that it is up to the scientific community to decide what is the appropriate cut-off for their application.

Rasmussen et al.^1^ highlight that there are some types of published data that we did not use in our study. These data include in silico ACE2 receptor/spike RBD interaction predictions^4–6^, in vitro surface plasmon resonance assays^7^, and in vitro analyses using cell lines^7^. Using these examples, Rasmussen et al.^1^ specifically refer to our prediction, at two of the three aforementioned cut-offs, that the European hedgehog may be susceptible to infection with SARS-CoV-2 (at 0.93 confidence, with an SD of ±0.23). While we agree that data produced from laboratory model studies, and structural and sequence information on ACE2 receptor orthologues and RBDs from hosts and virus strains, can provide insight into potential infectivity, information for the vast majority of interactions and ACE2 orthologues were (and still are) not available or determined for the overwhelming majority of hosts/coronaviruses (as mentioned in our discussion in ref. 2). Consequently, if we based our analysis on this interaction (or indeed on in silico predicted interaction from sequences), we would have been unable to include the vast majority of both coronaviruses and hosts in our study. Moreover, in the context of machine learning, sets of data (e.g. ACE2 orthologue sequences) which are only available for a small fraction of the potential hosts (and completely absent for the majority), cannot be incorporated into training and validation pipelines.

A large component of the argument proposed by Rasmussen et al.^1^, that the European hedgehog does not appear to be susceptible to SARS-CoV-2, is based on a lack of protein-cell (Spike RBD-ACE2 orthologue) interaction. One of the studies which they cite, Luan et al.^6^, used ACE2 sequence information to predict in silico binding to SARS-CoV-2 RBD, also predicted that the raccoon dog (*Nyctereutes procyonoides*) “could be ruled out from the potential host list for SARS-CoV-2”; subsequently, however, raccoon dogs were experimentally demonstrated as hosts of SARS-CoV-2^8^. This demonstrates the problem of ruling out one model’s predictions with the results of another; all models have a false-discovery rate (we clearly outline all available metrics of ours in our study and discuss below), and hence we believe that the results of all models should be published: it is then up to the scientific community to consider the merits and applicability of individual approaches to individual situations.

Accordingly, given the above limitations, the lack of ACE2/Spike RBD data for the vast majority of hosts and virus strains (as mentioned in Wardeh et al.^2^), and the logistic limitation of there being no large-scale repository of the data which do exist, they were not included in our study (again, as detailed in Wardeh et al.^2^). However, we do agree that these data could be used as a means of validating our results, albeit with a low degree of certainty and very low degree of coverage of the possible associations. Instead, we opted to compare our predictions to more recent field-observations that were published after our ‘observed interaction’ data used in the study were acquired; as stated in the discussion of Wardeh et al.^2^. We believed, and maintain, that these data are more suited to the purpose of validation as they show in situ field-observed associations, which is precisely what we aimed to predict. These data do have their own limitations, such as a lack of the ability to demonstrate ‘no-possible interaction’, and the smaller numbers of observed interactions, but we feel the applicability advantages outweigh these drawbacks.

We do of course accept that our methods can (and will) produce false-positive predictions. In the interests of transparency and to provide the scientific community with as much detail as possible on our models’ performance, we have presented^2^ (and in the contained supplementary information files of ref. 2): confusion matrices, AUC, TSS, and F-Scores, for our models, including at all three of the above cut-offs. This is in line with or exceeds all other studies in the field that we are aware of.

During the review process, the issue of: “Classifying virus-host interactions as either ‘observed’ or ‘unknown’ for training leaves no possibility for the algorithm to learn which interactions aren’t plausible.”, was raised. We agree with this criticism; however, no such repository of information (i.e. virus X cannot infect host Y) currently exists, and hence we, and indeed all other studies in the field e.g.^9,10^, are unable to include these ‘true negative’ associations in the way described by the reviewer. Addressing this issue and generating such a repository is a current focus of our ongoing work, and we hope to be able to include such ‘true negative’ associations in our future virus/host association work.

In summary, we thank Rasmussen et al.^1^ for their comments. Our reply provides an overview of the methods in a more accessible format to a general reader and discusses the issue of additional data derived from in silico and in vitro predictions (as opposed to in situ ‘field observations’). All parties seem in agreement that inclusion of these data in the model itself would be inappropriate, but that such data could or should be used to discuss the likely accuracy of the output. We believe it could, but given the vast number of predictions and lack of direct field-applicability of in silico and in vitro predictions, it is preferable to focus on *post hoc* field observations for discussion of field accuracy of our predictions. Nonetheless, we concede that this would have added to the discussion, and believe that the discussion here ameliorates this omission.

## Supplementary information

Supplementary Information

## Data Availability

No new data were generated for this reply. Please note that in their article, Rasmussen et al. claim to have been unable to find specific outputs of our pipelines; the link to all of this information can be found in the ‘Data availability’ of ref. 2, 10.6084/m9.figshare.13110896.
