# Supplementary material for: Reply to: Machine-learning prediction of hosts of novel coronaviruses requires caution as it may affect wildlife conservation
Source: Nat Commun. 2022 Sep 12;13:5102. doi: 10.1038/s41467-022-32747-6 (PMC9467978; doi:10.1038/s41467-022-32747-6)
Supplement: Supplementary file 1 — Supplementary Information [file 41467_2022_32747_MOESM1_ESM.pdf]

Reply to: Machine-learning prediction of hosts of novel coronaviruses requires caution as it may affect wildlife conservation

Supplementary Information

Marcus S.C. Blagrove*, Matthew Baylis, Maya Wardeh*

*Correspondence to: marcus.blagrove@liverpool.ac.uk, maya.wardeh@liverpool.ac.uk

Supplementary Table 1 – Learners, similarities and calculations computed in each of the three perspectives.

Perspective	Learners	Factors (similarities)	Vector representation (N = length of vector)	Calculation	SNF
Viral	Genomic distance	Genomic similarity	No	1-normalised hamming distance.	No
	Biases and codon usage	Biases and codon usage similarity	N=3,264 – nucleotide, dinucleotide, codon, and codon pair score (CPS) biases.	Cosine similarity between vectors	No
	Secondary structure (predicted)	Helix (H) – coverage	N = 100 – coverage for each 1% of the genome length (number of times a structure was predicted)		Yes
		Beta-Sheet (E) – coverage			
		Coil (C) – coverage			
		Helix (H) – probability	N = 100 – for each 1% of the genome length mean probability of the structure (in the percent of the genome considered)		
		Beta-Sheet (E) – probability			
		Coil (C) – probability			
Mammalian	Phylogenetic	Phylogenetic similarity	No	1- normalised phylogenetic distance.	No
	Life-history and reproductive traits	Life-history traits	N=4 - Body mass (g), maximum age (months) activity cycle (3 categories), migration (yes/no)	1- normalised Gower's distance.	Yes

		Reproductive traits	N = 6 - Age at sexual maturity (days), gestation period length (days), litters per year, litter size and weaning age (days)		
	Habitat	Habitat utilisation	N = 14 - binary indicators of whether a species uses one or more of 14 natural and artificial habitats		No
	Diet	Diet utilisation	N = 10 - Proportional use of 10 diet categories		
	Geographical Overlap	Geographical Overlap (yes/no)	NO	1 (there is overlap) or 0 (no overlap)	
	Climate	Mean temperature	N = 11 - Quantile vector representing the probabilities: 5%, 10%, 20%, 30%, 40%, 50%, 60%, 70%, 80%, 90%, and 95%.	Cosine similarity between vectors	Yes
		Mean precipitation			
	Geospatial	Evergreen/deciduous needle-leaf trees (%)			
		Evergreen broad-leaf trees (%)			
		Deciduous broad-leaf trees (%)			
		Mixed/other trees (%)			
		Shrubs (%)			
		Herbaceous vegetation (%)			
		Barren land (%)			
		Managed/Cultivated Vegetation (%)			
		Regularly flooded vegetation (%)			
		Cropland (%)			
		Pasture (%)			
		Livestock and poultry (head count for each included species)			
		Human population			
		Urban land (%)			

		Mammalian diversity - Number of different mammalian species in a grid cell.			
Network	Viral network similarity	Viral network similarity	N = 126 - Vector representation of each virus node using DeepWalk	Cosine similarity between vectors	No
	Mammalian network similarity	Mammalian network similarity	N = 126 - Vector representation of each mammalian node using DeepWalk		
